# Paraesophageal hernia repair with bifacial mesh

**Published:** 2016

**Authors:** S Ungureanu, N Șipitco, N Gladun, C Lepadatu

**Affiliations:** * Department of General Surgery, Republican Clinical Hospital, Chisinau, Moldova; ** Department of Surgery, Faculty of CME, „Nicolae Testemitanu” State Medical and Pharmaceutical University, Chisinau, Moldova

**Keywords:** hiatus hernia, paraesophageal hernia, type II hiatus hernia, surgical mesh

## Abstract

Abstract

**Background.**The paraesophageal hiatus hernias (PHH) are relatively uncommon, but an increased incidence has been reported and they now account for 5–10% of all hiatus hernias. The surgical treatment is recommended for all the patients with this pathology because of high risk of complications: obstruction, incarceration, strangulation or perforation. The use of prostheses is recommended in the process of repairing the giant PHH because the main problem of this operation is the high rate of recurrence.

**Case presentation.**The patient is a 44-year-old male with a large and symptomatic paraesophageal hernia. Diagnosis was confirmed by instrumental examination. An elective laparoscopic repair was carried out by using polypropylene bifacial anti adhesive synthetic mesh (Surgimesh XB Aspide Medical). The postoperative period passed without severe complications.

**Conclusions.**The laparoscopic approach as a therapeutic option can be successfully used in the repair of paraesophageal hernia. A selective use based on clinical experience was recommended, as the technique appeared to be safe, and in case of large hiatus hernia with hiatal defect, greater than 5 cm, the application of synthetic material to minimize the recurrence rate was recommended.

## Introduction

It is estimated that 10-15% of the general population have hiatus hernias, of which more than 90% are sliding hiatus hernias (Type I) [**1**]. 

In paraesophageal hiatus hernias (PHH) Type II, the gastroesophageal junction remains in its normal anatomic position, but the fornix of the stomach herniates into the thorax anterior to the esophagus and within a hernia sac of peritoneum. Approximately 50% of the patients with PHH are asymptomatic or complain of only minor symptoms [**2**]. Characteristic clinical signs of PHH include chest pain, postprandial distress (epigastric fullness, nausea, intermittent vomiting, chest discomfort, and dyspnea). Symptoms of gastroesophageal reflux are not typical for hernias of type II. In as many as 20% of the patients, the clinical presentation of a massive and incarcerated PHH may be urgent or emergent. Therefore, in the absence of restrictive operative risk, all the symptomatic patients should undergo elective repair to prevent life-threatening complications, such as obstruction, strangulation, perforation and bleeding. 

The surgical treatment of PHH is currently the only effective therapy. The traditional repair of PHH can be done by left thoracotomy or laparotomy. The minimally invasive laparoscopic approach has been the gold standard in the treatment of hiatus hernias over the past decade [**3**,**4**]. Untreated hiatal hernia with gastroesophageal reflux disease is an important causal factor, which defines a specific group of patients at risk to develop Barrett esophagus and esophageal adenocarcinoma [**5**]. The laparoscopic repair follows the same surgical principles adopted in the traditional operation. Prostheses are recommended in the repair of giant hiatus hernia with hiatal defect greater than 5 cm. Repair with synthetic mesh has the potential of reducing the recurrence rate [**6**,**7**,**8**]. The experience of our department began in 2003, by which time we had accumulated more than 9 years of standing, performing laparoscopic repair for gastroesophageal reflux due to Type I hiatus hernias (120 cases). The laparoscopic solution for paraesophageal hernias was applied to 12 patients, 4 of them with mesh repair. 

The article describes the clinical case of laparoscopic bifacial mesh repair of giant paraesophageal hernia with good distant results.

## Case presentation

The patient was Mr. B, a 44-year-old male with a large and symptomatic Type II (paraesophageal) hernia. He presented the following clinical symptoms: chest pain, postprandial epigastric fullness, chest discomfort and dyspnea. As described in the patient’s own words, these symptoms appeared 2 years before. During 2 years, the patient used symptomatic conservative treatment without any essential improvement. Routine computer tomography revealed a large, 130 x 80 mm cavitary mass with an aerogenic component situated in the posterior mediastinum. 

**Fig 1:**
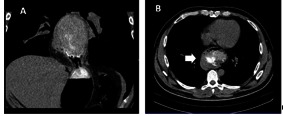
**Fig 1 a,b**CT cavitary aerogenic mass situated in posterior mediastinum (arrow) (frontal and transversal view)

Barium swallow study showed a large hiatus hernia type II, more than 1/ 3 of the stomach, situated above the diaphragm, and revealed the presence of high gastroesophageal reflux. 

**Fig 2:**
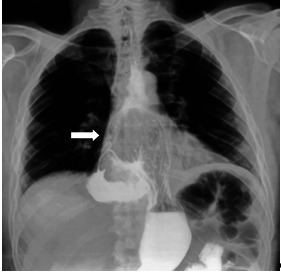
**Fig 2**Barium swallow study showing a giant type II paraesophageal hiatus hernia. More than 1/ 3 of the stomach is above the diaphragm (arrow)

Fibrogastroscopy detected insufficiency of cardia, reflux esophagitis gr. 1 (without clinical symptoms of reflux) and more then 1/ 3 of stomach situated above the diaphragm.

**Fig 3:**
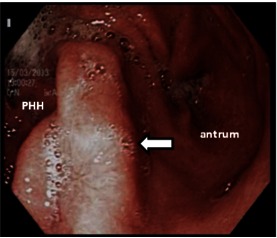
**Fig 3**Endoscopic picture – more than 1/ 3 of the stomach is above the large diaphragmatic opening (arrow)

An elective laparoscopic repair was carried out on March 28, 2013. Abdominal inspection detected a large paraesophageal hernia of more than 1/ 3 of the stomach, situated above the diaphragm. Dissection and removal of the hernia’s peritoneal sac from the mediastinum was performed to minimize the risk of recurrence. Repair of the diaphragmatic crura with interrupted permanent sutures was followed by anterior diaphragmoraphy in order to considerably reduce the enlarged hiatus. The bifacial silicon mesh was installed around the esophagus on the diaphragmatic crura and fixed with several separated sutures. The surgical intervention was finished with complete fundoplication (Nissen-Rossetti procedure) and gastropexy. The postoperative period passed without severe complications and the patient was discharged in 3 days. The common postoperative symptom as dysphagia appeared, but it was very mild and had resolved within two weeks by conservative treatment. Barium swallow study was done in a month after the surgical intervention, and showed the normal position of stomach under the diaphragm. One-year postoperative follow-up showed no recurrence of complications.

**Fig 4:**
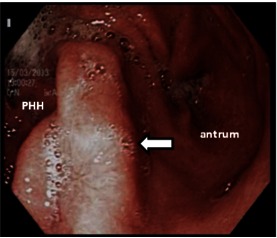
**Fig 4**Barium swallow study showing the normal position of the stomach in a month after the surgical intervention

## Discussion

All our patients with hiatus hernias were symptomatic, and surgical intervention was clearly indicated to relieve the symptoms and to prevent the development of potentially serious complications. In this case report, the patient was symptomatic with impact on the quality of life. The use of prostheses is recommended in the repair of giant hiatus hernia with hiatal defect greater than 5 cm. The information currently available shows that the use of a mesh for hiatal repair is safe and prevents recurrence [**8**,**9**]. Frantzides et al. showed the results of a prospective randomized trial comparing the simple closure with polytex onlay reinforcement for paraesophageal hernia repair, in cases with a hiatus wider than 8 cm. Recurrences were significantly reduced after mesh placement (20% vs. 0%; P<.001), without any long-term sequel, after a 40-month follow-up period [**10**]. The main problem with the use of mesh in the hiatus is the risk of local complications (fibrosis and adhesions, erosion, or perforation). Nonetheless, the incidence of mesh-related complications in the hiatus is currently less than 2%, although no reports on long-term outcome (>10 years) are available [**11**]. There are different types of prostheses used in the repair of hernias: polypropylene, polytetrafluoroethylene (PTFE), SIS (small intestinal submucosa), polyglycolic acid/ trimethylene carbonate mesh, biomesh (Strattice™, Alloderm™, Surgisis™, Permacol™) [**12**,**13**].

In 2008, GORE introduced BIO-A tissue reinforcement, an absorbable mesh that acts as a scaffold for cells to lay down new matrix material as it is absorbed [**13**]. This may be advantageous in avoiding complications such as erosion and infection by minimizing foreign body presence. Synthetic meshes can provoke a foreign body response that results in the encapsulation and can cause long-term infection and extrusion of the implant. The acellular dermal matrix leaves the structural architecture intact and provides scaffolding for tissue growth with continued reinforcement. The absorbable mesh is accepted by the body without an immunological or foreign body response [**14**]. 

Several studies have shown that laparoscopic hiatal hernia repair is associated with high recurrence rates of up to 42% as a result of the difficulty in the closure of the hiatal gap and demonstrated advantages of open techniques vs. minimally invasive approach [**11**,**15**]. Some authors consider that the use of prostheses is unreasonable because of local complications and high rate of recurrence [**16**,**17**].

However, starting with 2010, the surgical mesh was applied in 7 cases of laparoscopic hiatus hernia repair in our department, 4 of them being patients with paraesophageal hernias. Polypropylene bifacial anti adhesive synthetic mesh (Surgimesh XB) was used in all cases with good results, without severe complications. In the presented case report of giant paraesophageal hernia, the use of bifacial silicon-coated mesh led to good results confirmed by the clinical and instrumental examination. 

## Conclusions

The laparoscopic approach as a therapeutic option can be successfully used in the repair of giant paraesophageal hernia. A selective use based on clinical experience is recommended, as the technique appears to be safe, and in case of large hiatus hernia with hiatal defect greater than 5 cm, the application of synthetic material to minimize recurrence rate is recommended. The surgical mesh with anti adhesive properties should be used in order to minimize the eventual complications.

### Disclosures

None
